# Migraine and Restless Legs Syndrome: A Meta‐Analysis

**DOI:** 10.1111/jsr.70202

**Published:** 2025-09-19

**Authors:** Florindo d'Onofrio, Maria Cropano, Giada Panzino, Mariachiara Gaita, Giulio Cicarelli, Piero Barbanti, Gerardo Casucci, Simona Raimo, Antonio Costanzo

**Affiliations:** ^1^ Stroke Unit San Giuseppe Moscati, Hospital Avellino Avellino Italy; ^2^ UOSD Second Neurology University of Campania ‘Luigi Vanvitelli’ Naples Italy; ^3^ Department of Health Sciences Magna Graecia University of Catanzaro Catanzaro Italy; ^4^ Department of Medical and Surgical Sciences Magna Graecia University of Catanzaro Catanzaro Italy; ^5^ Department of Psychology University of Campania ‘Luigi Vanvitelli’ Caserta Italy; ^6^ Neurology Unit San Giuseppe Moscati, Hospital Avellino Avellino Italy; ^7^ IRCCS San Raffaele Rome Italy; ^8^ San Raffaele University Rome Italy; ^9^ San Francesco Hospital‐Telese Terme Telese Terme Italy; ^10^ Department of Humanities University of Federico II Naples Italy

**Keywords:** meta‐analysis, migraine, prevalence, restless legs syndrome, sleep

## Abstract

Restless legs syndrome is a sensorimotor disorder of sleep/wake regulation that frequently coexists with migraine, affecting patients' quality of life. This study aimed to estimate the prevalence of restless legs syndrome in individuals with migraine and to explore the associated clinical, demographic and behavioural aspects. A systematic review and meta‐analysis of the existing literature was conducted. Articles were included if they provided data on individuals with migraine, with or without restless legs syndrome and these proportions were used to estimate the prevalence of restless legs syndrome in migraine. A total of 30 studies were included in the meta‐analysis. The overall pooled prevalence of restless legs syndrome among individuals with migraine was 20% (95% confidence interval [CI] = 17%–23%). The pooled prevalence of restless legs syndrome was affected by migraine duration (higher prevalence with longer duration), disability (higher prevalence with greater migraine‐related disability) and age (higher prevalence in older individuals). A significant association was found between restless legs syndrome, migraine with aura and chronic migraine. No significant sex‐related differences were observed. Among behavioural factors, depression, pain and poor sleep quality were significantly associated with restless legs syndrome. These findings suggest that restless legs syndrome is a common comorbidity in migraine with aura and chronic migraine, with a higher prevalence than that reported in the general population. Moreover, specific demographic, clinical and behavioural characteristics may help identify individuals at higher risk. Further studies are needed to clarify the underlying pathophysiological mechanisms and to identify potential therapeutic targets.

## 1 Introduction

Migraine is a severe, chronic and disabling neurological disorder, typically characterised by recurrent attacks of moderate‐to‐severe unilateral headache, often accompanied by nausea, vomiting, photophobia, phonophobia and hypersensitivity of various central nervous functional systems (Goadsby et al. 2017; Schürks et al. 2014). It is one of the most prevalent neurological conditions worldwide, affecting approximately 12%–15% of the general population, with a higher prevalence among women than men (Al‐Hassany et al. 2020; Lipton et al. 2001).

Beyond its characteristic headache episodes, migraine has been increasingly recognised as a complex brain disorder associated with multiple comorbid conditions, including psychiatric disorders (e.g., depression, anxiety), chronic pain syndromes (e.g., fibromyalgia, temporomandibular disorder) and sleep disturbances (Diener et al. 2008; Rains 2018). Among the latter, restless legs syndrome (RLS) has gained particular attention due to its frequent co‐occurrence with migraine and the potential shared pathophysiological mechanisms underlying both disorders (Allen et al. 2005; Walters et al. 2004). RLS is a common neurological sensory‐motor disorder characterised by an irresistible urge to move the legs, typically accompanied by uncomfortable or unpleasant sensations, mainly affecting the ankles and calves. Symptoms tend to worsen during periods of rest or inactivity, especially in the evening and during the onset of sleep and are partially or completely relieved by movement (Allen et al. 2005). RLS prevalence in the general population is estimated at 5%–10%, with a higher frequency in women and older individuals (Manconi et al. 2012; Seeman 2020).

Several studies have reported an increased prevalence of RLS in individuals with migraine, suggesting a bidirectional relationship between the two conditions (Mail Gurkan et al. 2022; Schürks et al. 2014). The pathophysiological mechanisms linking migraine and RLS are not fully understood but may involve common neurobiological pathways, including dopaminergic dysfunction, iron metabolism disturbances and genetic predisposition (Paulus et al. 2007; Sabayan et al. 2007; Trenkwalder and Paulus 2010). Specifically, alterations in dopaminergic transmission have been implicated in both disorders, with evidence suggesting that hypersensitivity of the dopaminergic system may contribute to migraine pathophysiology and RLS symptoms (Guo et al. 2017). Moreover, iron deficiency, which plays a crucial role in dopamine synthesis and function, has been observed in both migraine and RLS, further supporting a potential link between these conditions (Cameli et al. 2023).

Neuroimaging and electrophysiological studies have also provided insights into the shared neural substrates of migraine and RLS. Functional magnetic resonance imaging (fMRI) studies suggest that alterations in sensorimotor and pain‐processing networks may contribute to the sensory disturbances observed in both disorders (Sarıcam and Sarıcam 2021; Schulte et al. 2015). Additionally, genetic studies have identified overlapping risk loci associated with migraine and RLS, indicating a possible common genetic predisposition (Anttila et al. 2018).

From a clinical perspective, the co‐occurrence of migraine and RLS may have significant implications for disease burden and patient management. Individuals suffering from both conditions often experience poorer sleep quality, higher levels of disability and reduced quality of life compared to those with either disorder alone (Ferini‐Strambi et al. 2019). Furthermore, certain medications commonly used for migraine prophylaxis, such as dopamine agonists and beta‐blockers, may influence RLS symptoms, necessitating careful therapeutic considerations in individuals with both conditions (Trenkwalder et al. 2016).

Given the growing body of literature on the association between migraine and RLS, further research is needed to clarify the underlying mechanisms and identify potential therapeutic targets. A previous systematic review of 15 studies highlighted the high prevalence of RLS in migraine patients and the need for additional studies to better understand demographic, clinical and neurobiological aspects influencing this association (Wang et al. 2019). Building upon this foundation, we conducted an updated systematic review and meta‐analysis to provide a comprehensive assessment of the current evidence on the relationship between migraine and RLS, with the aim of elucidating the potential mechanisms driving their co‐occurrence and informing clinical practice.

## 2 Methods

### 2.1 Study Design

A systematic literature search was performed and updated up to January 2025 using PubMed, Scopus and PsycINFO (PROQUEST) databases and entering the following search terms: (migraine OR migraineurs OR headache) AND (‘restless legs syndrome’ OR RLS OR ‘movement‐sleep disorders’). After that, a manual search of references of included studies and reviews was conducted to find more relevant articles. The literature search was conducted independently by two reviewers (S.R., G.P.). This systematic review was conducted following the Preferred Reporting Items for Systematic Reviews and Meta‐Analyses (PRISMA) statement (Moher et al. 2009, 2015) and preregistration of the review protocol was undertaken electronically on the PROSPERO International prospective register of systematic reviews (CRD420250646471).

### 2.2 Study Selection

Two independent observers (G.P., M.C.) examined the results from the literature and each case of disagreement was resolved by resorting to a third arbitrator (S.R.).

Studies were eligible for inclusion in this review if they: (a) were published in peer‐reviewed journals in English; (b) investigated the association between migraine and RLS in an adult sample (i.e., ≥ 18 years); (c) provided statistical results (i.e., mean, standard deviation and *p*‐value) for comparisons of demographic, clinical and behavioural measures between individuals with migraine, with and without RLS (respectively Migraine + RLS or Migraine − RLS).

The diagnostic criteria for RLS established by the International RLS Study Group (IRLSSG) define the condition based on the presence of a compelling urge to move the legs, which typically occurs during rest or inactivity, especially in the evening or nighttime and is either partially or fully alleviated by movement (Allen et al. 2014). In contrast, the criteria set out by the International Classification of Sleep Disorders—Third Edition (ICSD‐3) include an additional requirement: the presence of significant clinical distress to confirm the diagnosis (Sateia 2014).

During the initial phase of the selection process, we excluded review articles, animal studies, non‐English language publications, conference proceedings, theses, case reports, editor letters, commentaries and studies unrelated to the specific topic of interest.

We included studies with the biggest sample when two or more studies provided information obtained from the same database/sample. Studies that explicitly reported matched‐size groups were excluded from the analysis of the prevalence of RLS because of their effects that distort the overall prevalence outcomes.

Quality assessment was conducted using the modified version of the Quality Assessment of Diagnostic Accuracy Studies (QUADAS; Broen et al. 2012, 2016) tool, a widely recognised instrument that is particularly appropriate for prevalence studies. A score of 14 or higher indicates acceptable quality.

The two reviewers initially evaluated the eligibility of the identified studies by independently reviewing titles and abstracts. Articles deemed potentially eligible were then read in full by both reviewers. Any discrepancies between them were revisited and, if necessary, resolved through discussion with a third reviewer until a consensus was reached.

### 2.3 Outcomes

We considered the prevalence of RLS in individuals with migraine as the main outcome and the following demographic, clinical and behavioural features associated with its occurrence: (a) demographic aspects: age (years) and sex (codified as the proportion of males); (b) migraine‐related variables: age at migraine onset, disease duration (years from migraine diagnosis), migraine disability (Migraine Disability Assessment [MIDAS]; Headache Impact Test [HIT‐6]), migraine clinical subtype (migraine with aura [MA]; migraine without aura [MwoA]; chronic migraine [CM]) and (c) neuropsychiatric and behavioural aspects: depression, anxiety, sleepiness, pain (evaluated by means and standard deviation of screening questionnaires).

### 2.4 Data Extraction

Two reviewers (S.R., G.P.) independently extracted and summarised data from the included studies, using a standardised form that included the following: (a) characteristics of the publication: name of the first author, year of publication and country; (b) characteristics of the individuals with Migraine + RLS or Migraine − RLS: including sample size, demographic aspects and RLS‐related clinical and behavioural variables.

Specifically, demographic, clinical and behavioural measures were extracted as reported in the primary studies and refer to the entire sample of individuals with Migraine + RLS or Migraine − RLS.

Moreover, in studies reporting on CM, the presence or absence of aura was not specified; therefore, we assumed that the data mainly referred to MwoA, given the lower prevalence of CM among individuals with MA (Domitrz and Chądzyński 2025; Tsao et al. 2022).

We considered the proportion of participants diagnosed with RLS and the total number of included patients for each study population as an outcome for the prevalence meta‐analysis, whereas we extracted statistical indices (mean and standard deviation or *p* values) to compute effect sizes (ESs) of comparisons on demographic, clinical and behavioural measures between individuals with Migraine + RLS and Migraine − RLS.

### 2.5 Statistical Analysis

Pooled prevalence and 95% confidence intervals (95% CIs) were calculated using the proportion of participants diagnosed with RLS (Migraine + RLS) and the total number of patients included in each study population as variables. Moreover, we performed several meta‐regressions to further explain heterogeneity across study findings when there were at least 10 samples to 1 covariate (Borenstein et al. 2009); we explored the possible influence on RLS prevalence in migraine of clinical variables (i.e., age at migraine onset, migraine duration, MA subtype, MwoA subtype, CM subtype, migraine disability, frequency of migraine attacks); demographic variables (i.e., age and sex); and behavioural variables (i.e., depression, anxiety, sleep and pain).

To investigate the association between RLS and demographic, clinical and behavioural aspects reported as continuous variables, we computed the ESs from data reported in the primary studies by comparing data from the Migraine + RLS and Migraine − RLS groups using Hedges' g unbiased approach (Cohen 1988). Then, we defined values < 0.20 as small effects, values of about 0.50 as moderate effects and values of approximately 0.80 as large effects. The associations between the occurrence of RLS and variables reported as categorical variables (frequencies and percentages) such as sex, migraine subtype MA, MwoA and CM subtypes were evaluated using risk ratio (RR).

Random‐effects models were used to estimate the meta‐analytic results and test whether the mean ES was significantly different from zero (*p* < 0.05, two‐tailed). Heterogeneity among the studies was evaluated using the *Q* and *I* ^2^ statistics index (Huedo‐Medina et al. 2006). Egger's regression test (Egger et al. 1997) and the trim and fill method (Duval and Tweedie 2000) were used to explore the possible presence of publication bias, whereas sensitivity analyses using the leave‐one‐out method were performed to identify studies that produced a disproportionate influence on the overall results. This approach is in line with other recent meta‐analyses applying similar methodological frameworks to neurological and cognitive domains (Raimo et al. 2021, 2024). Statistical analyses were performed using the meta‐analytic software, ProMeta 3.0.

## 3 Results

### 3.1 Literature Search and Study Selection

**FIGURE 1:**
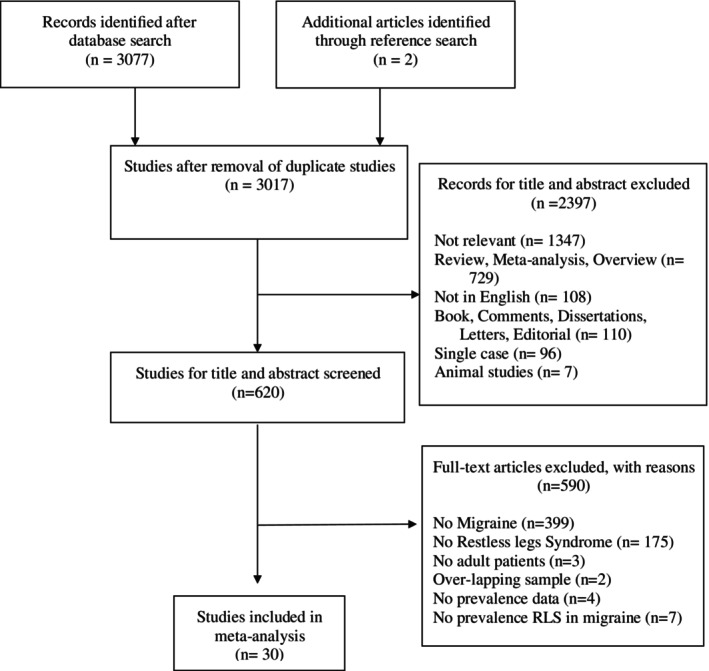
Flowchart of the selection process of primary studies.

Figure 1 summarises the study selection process. A total of 3079 articles were identified by the literature search. After eliminating duplicates and title and abstract reading, 620 potential articles remained for full‐text screening. Of the 620 articles, 590 were excluded because they did not fulfil the inclusion criteria. Thirty studies obtained a score above the QUADAS cut‐off (≥ 14) and were thus included in the meta‐analysis (QUADAS assessment was reported in Supporting Information Data S1).

### 3.2 Study Characteristics

The demographic and clinical characteristics of the 30 included studies are summarised in Table 1, while behavioural characteristics are detailed in Supporting Information Data S2. A total of 20,781 participants were analysed across studies, including 3343 individuals with Migraine + RLS (106 males) and 17,438 individuals with Migraine − RLS (910 males). The mean age of the Migraine + RLS group was 38.75 years (SD = 10.04), whereas that of the Migraine − RLS group was 36.92 years (SD = 10.7). The average years of education were 11.69 (SD = 2.32) for the Migraine+RLS group and 11.06 (SD = 2.19) for the Migraine − RLS group.

**TABLE 1:** Characteristics of studies investigating RLS in migraine.

Authors	Country	Migraine + RLS patients									
		*n*	Age	nM	Edu	Age migraine onset	Migraine duration (years)	Migraine clinical subtype	Migraine disability	Attack frequency (month)	Treatment
Acar et al. (2016)	Turkey	27	NR	NR	NR	NR	8.9 ± 6.7	MA (20) MwoA (7)	0.80 ± 0.15 (MIDAS)	NR	NO
Akdag Uzun et al. (2018)	Turkey	66	37.36 ± 9.39	NR	NR	11.39 ± 7.66	NR	MA (21)	NR	NR	NR
Aldemir et al. (2020)	NR	18	34.5 ± 1.8	0	9.89 ± 1.23	24.94 ± 1.91	6.90 ± 1.17	MA (13) MwoA (5)	NR	9.84 ± 2.31	YES NSAIDs Paracetamol Acetylsalicylic acid
Chen et al. (2010)	Taiwan	88	40.1 ± 12.7	12	NR	NR	NR	MA (4) MwoA (29) CM (49)	42.9 ± 52.2(MIDAS)	19.52 ± 10	YES (64) TCA Dopamine antagonists MirtazapineSSRIs or SNRIs
Chen et al. (2016)	Taiwan	30	35.5 ± 9	2	NR	NR	NR	CM(8)	NR	NR	NO
Cho et al. (2015)	Korea	13	39.8 ± 12.6	3	NR	NR	NR	NR	59.1 ± 9.4 (HIT‐6)	5.8 ± 8.6	NR
Cologno et al. (2008)	Italy	42	NR	7	NR	NR	NR	NR	NR	NR	NO
D'Onofrio et al. (2008)	Italy	44	NR	7	NR	NR	NR	NR	NR	NR	NR
D'Onofrio et al. (2011)	Italy	6	38.2 ± 6.6	3	NR	20.2 ± 8.7	NR	MA	NR	0.28 ± 0.08	NR
Ferreira et al. (2013)	Brasil	18	NR	NR	NR	NR	NR	NR	NR	NR	NR
Fuh et al. (2016)	Taiwan	211	39.2 ± 13.3	38	NR	NR	NR	MA (29) MwoA (182) CM(95)	38.1 ± 54.8 (MIDAS)	12.5 ± 9.7	YES (46)
Jiang et al. (2022)	Taiwan	264	NR	NR	NR	NR	NR	NR	NR	NR	NR
Karthik et al. (2012)	India	29	NR	NR	NR	NR	NR	NR	NR	NR	NR
Karthik and Patil (2019)	India	27	32.41 ± 8.78	7	NR	NR	NR	MwoA	14.04 ± 11.36 (MIDAS)	NR	YES (3) Flunarizine
Lin et al. (2016)	Taiwan	24	NR	NR	NR	NR	NR	MA (12) MwoA (12)	NR	NR	NR
Lin et al. (2020)	Taiwan	53	43.78 ± 11.01	11	13.49 ± 3.41	NR	NR	MA (26) MwoA (27) CM (20)	23.40 ± 19.18 (MIDAS)	10.63 ± 7.31	NR
Lucchesi et al. (2012)	Italy	63	NR	NR	NR	NR	NR	NR	NR	NR	NO
Mail Gurkan et al. (2022)	Japan	17	37.17 ± 7.38	3	NR	NR	5.29 ± 5.39	MA (10) MwoA (7) CM (3)	NR	9.64 ± 4.48	NO
Muayqil et al. (2018)	Saudi Arabia	386	NR	NR	NR	NR	NR	MA (154) MwoA (235)	NR	NR	NR
Rhode et al. (2007)	Germany	71	47.1 ± 11.8	NR	NR	NR	21.9 ± 12.5	NR	NR	3.8 ± 4.1	YES
Schurks et al. (2012)	USA	996	NR	0	NR	NR	NR	NR	NR	NR	NR
Suzuki et al. (2011)	Japan	36	37.0 ± 12.7	4	NR	NR	16.1 ± 9.6	MA (9) CM (10)	30.0 ± 37.0 (MIDAS)	NR	YES (4) Triptans Analgesic
Suzuki et al. (2021)	Japan	21	NR	NR	NR	NR	NR	NR	NR	NR	YES Antidepressants Antiepileptics Others
Suzuki et al. (2024)	Japan	55	NR	NR	NR	NR	NR	NR	NR	NR	YES Antidepressants Antiepileptics
Valente et al. (2017)	Italy	29	46.1 ± 12.4	5	NR	NR	NR	MA (12)	NR	8.1 ± 9.5	YES SNRI (1) SSRI (3) TCA (2) SARI (1) NSAID (23) Triptans (10) Opiates (1) Anticonvulsant (3) Benzodiazepine (7) Estroprogestinic (4)
Van Oosterhout et al. (2016)	Netherlands	403	NR	NR	NR	NR	NR	MA (170) MwoA (233)	NR	NR	NR
Winter et al. (2013)	France	245	NR	NR	NR	NR	NR	NR	NR	NR	NR
Yang et al. (2018)	Taiwan	22	33.0 ± 9.0	2	NR	NR	14.5 ± 10.8	MA (11) MwoA (11)	NR	7.8 ± 6.3	YES (2) Ropinirolo
Yang et al. (2019)	Taiwan	22	41.4 ± 12.7	1	NR	NR	6.4 ± 6.7	MA (5) MwoA (17)	NR	11.2 ± 9.5	NR
Young et al. (2003)	USA	17	37.41 ± 9.52	1	NR	NR	NR	NR	NR	NR	YES DRBA (13) Anticholinergic (3) Benzodiazepine (5) SSRI (8) Opiate (3) Gabapentin (1) Calcium channel blocker (2) β‐blocker (2)

Authors	Country	Migraine − RLS patients										RLS assessment	QS
		*n*	Age	nM	Edu	Age migraine onset	Migraine duration (years)	Migraine clinical subtype	Migraine disability	Attack frequency (month)	Treatment		
Acar et al. (2016)	Turkey	32	NR	NR	NR	NR	6.7 ± 4.9	MA (4) MwoA (28)	0.35 ± 0.06 (MIDAS)	NR	NO	IRLSSG	18
Akdag Uzun et al. (2018)	Turkey	134	31.51 ± 8.54	NR	NR	8.89 ± 7.66	NR	MA (15)	NR	NR	NR	IRLSSG	17
Aldemir et al. (2020)	NR	19	31.9 ± 1.6	0	8.00 ± 1.21	23.61 ± 2.22	10.94 ± 2.17	MA (9) MwoA (10)	NR	10.61 ± 2.64	YES NSAIDs Paracetamol Acetylsalicylic acid	IRLSSG	17
Chen et al. (2010)	Taiwan	684	42.4 ± 13.4	128	NR	NR	NR	MA (44) MwoA (238) CM (365)	30.8 ± 39.6 (MIDAS)	17.79 ± 10.43	YES (64) TCA Dopamine antagonists MirtazapineSSRIs or SNRIs	IRLSSG	18
Chen et al. (2016)	Taiwan	390	NR	NR	NR	NR	NR	NR	NR	NR	NO	IRLSSG	18
Cho et al. (2015)	Korea	130	41.5 ± 12.5	33	NR	NR	NR	NR	53.7 ± 9.2 (HIT‐6)	3.7 ± 6.0	NR	Questionnaire	16
Cologno et al. (2008)	Italy	122	37.1 ± 10.8	34	NR	21.3 ± 7.7	NR	MA (10) MwoA (114) Mixed (40)	NR	NR	NO	IRLSSG	17
D'Onofrio et al. (2008)	Italy	156	NR	44	NR	NR	NR	NR	NR	NR	NR	IRLSSG	18
D'Onofrio et al. (2011)	Italy	57	38.2 ± 14.7	NR	NR	22.6 ± 12.1	NR	MA	NR	0.81 ± 1.28	NR	IRLSSG‐R	18
Ferreira et al. (2013)	Brasil	54	NR	NR	NR	NR	NR	NR	NR	NR	NR	IRLSSG	17
Fuh et al. (2016)	Taiwan	781	39.2 ± 12.7	169	NR	NR	NR	MA (91) MwoA (690) CM (287)	22.3 ± 30.3 (MIDAS)	10.1 ± 8.6	YES (157)	IRLSSG	17
Jiang et al. (2022)	Taiwan	1383	NR	NR	NR	NR	NR	NR	NR	NR	NR	IRLSSG	15
Karthik et al. (2012)	India	61	NR	NR	NR	NR	NR	NR	NR	NR	NR	NCSDQ	17
Karthik and Patil (2019)	India	173	31.91 ± 10.0	49	NR	NR	NR	MwoA	11.54 ± 9.63 (MIDAS)	NR	YES (27) Amitriptyline (14) Flunarizine (9) Propranolo, topiramate, divalproex sodium (2)	IRLSSG	19
Lin et al. (2016)	Taiwan	348	NR	NR	NR	NR	NR	MA (99) MwoA (249)	NR	NR	NR	IRLSSG	18
Lin et al. (2020)	Taiwan	180	40.06 ± 11.37	27	14.11 ± 3.17	NR	NR	MA (34) MwoA (142) CM (51)	23.69 ± 16.22 (MIDAS)	8.78 ± 6.12	NR	IRLSSG	18
Lucchesi et al. (2012)	Italy	214	NR	NR	NR	NR	NR	NR	NR	NR	NO	IRLSSG	18
Mail Gurkan et al. (2022)	Japan	41	35.48 ± 8.17	6	NR	NR	8.21 ± 7.81	MA (25) MwoA (16) CM (7)	NR	7.51 ± 4.98	NO	IRLSSG‐II	16
Muayqil et al. (2018)	Saudi Arabia	947	30.99 ± 10.76	338	NR	NR	NR	MA (486) MwoA (833)	NR	NR	NR	Questionnaire	15
Rhode et al. (2007)	Germany	340	40.7 ± 12.4	NR	NR	NR	18.0 ± 11.9	NR	NR	3.2 ± 3.3	YES	Questionnaire	16
Schurks et al. (2012)	USA	5861	NR	0	NR	NR	NR	NR	NR	NR	NR	IRLSSG	16
Suzuki et al. (2011)	Japan	226	38.4 ± 13.0	43	NR	NR	17.9 ± 12.8	MA (58) CM (55)	17.1 ± 24.8 (MIDAS)	NR	YES (23) Triptans Analgesic	IRLSSG	17
Suzuki et al. (2021)	Japan	165	NR	NR	NR	NR	NR	NR	NR	NR	YES Antidepressants Antiepileptics Others	IRLSSG	18
Suzuki et al. (2024)	Japan	160	NR	NR	NR	NR	NR	NR	NR	NR	YES Antidepressants Antiepileptics	IRLSSG	17
Valente et al. (2017)	Italy	151	41.0 ± 12.8	32	NR	NR	NR	MA (42)	NR	6.6 ± 6.2	YES SNRI (2) SSRI (3) TCA (10) SARI (2) NSAID (116) Triptans (46) Opiates (5) Anticonvulsant (9) Benzodiazepine (13) Estroprogestinic (24)	IRLSSG	18
Van Oosterhout et al. (2016)	Netherlands	1981	NR	NR	NR	NR	NR	NR	NR	NR	NR	Questionnaire	17
Winter et al. (2013)	France	2571	NR	NR	NR	NR	NR	NR	NR	NR	NR	LSSG	15
Yang et al. (2018)	Taiwan	22	32.4 ± 7.7	3	NR	NR	11.9 ± 7.4	MA (11) MwoA (11)	NR	6.1 ± 5.8	NO	IRLSSG	17
Yang et al. (2019)	Taiwan	22	36.8 ± 9.9	4	NR	NR	6.7 ± 7.8	MA (4) MwoA (18)	NR	8.9 ± 4.8	NR	IRLSSG	17
Young et al. (2003)	USA	33	38.15 ± 11.55	6	NR	NR	NR	NR	NR	NR	YES DRBA (18) Anticholinergic (5) Benzodiazepine (8) SSRI (6) Opiate (3) Gabapentin (7) Calcium channel blocker (7) β‐blocker (2)	IRLSSG	17

Abbreviations: CM = chronic migraine; DRBA = dopamine receptor blocking agent; Edu = education; HIT‐6 = Headache Impact test‐6; IRLSSG = International Restless Legs Syndrome Study Group; MA = migraine with aura; MIDAS = migraine disability assessment; Migraine + RLS = patients with migraine and RLS; Migraine − RLS = patients with migraine without RLS; Mixed = migraine with and without aura; MwoA = migraine without aura; *n* = number of participants; NCSDQ = NIMHANS comprehensive sleep disorders questionnaire; nM = number of males; NR = not reported; NSAIDs = non‐steroidal anti‐inflammatory drugs; QS = quality score; RLS = restless legs syndrome; SARI = serotonin antagonist and reuptake inhibitors; SNRI = serotonin‐norepinephrine reuptake inhibitor; SSRI = selective serotonin reuptake inhibitor; TCA = tricyclic antidepressants.

Migraine subtypes were distributed as follows: in the Migraine + RLS group, 502 had migraine with MA, 792 with MwoA and 185 CM. In the Migraine − RLS group, there were 989 with MA, 2522 with MwoA, 765 with CM and 40 individuals with mixed‐type (i.e., with and without aura) migraine.

Regarding clinical variables, the average disease duration was 11.43 years (SD = 7.55) for Migraine + RLS and 11.48 years (SD = 7.83) for Migraine − RLS. The mean age of migraine onset was 18.84 years (SD = 6.09) in the Migraine + RLS group and 19.1 years (SD = 7.42) in the Migraine − RLS group. The average migraine disability score was higher in the Migraine + RLS group (29.76, SD = 26.29) compared to the Migraine − RLS group (22.78, SD = 18.54).

Attack frequency (mean number of attacks per month) was also elevated in the Migraine + RLS group (9.01, SD = 6.53) relative to the Migraine − RLS group (7.65, SD = 5.47).

In most studies, the presence of RLS was determined according to the IRLSSG diagnostic criteria. However, four studies (Cho et al. 2015; Muayqil et al. 2018; Rhode et al. 2007; Van Oosterhout et al. 2016) applied ad hoc questionnaires and one study (Karthik et al. 2012) used the NIMHANS Comprehensive Sleep Disorders Questionnaire (NCSDQ).

### 3.3 Meta‐Analytic Results on Migraine + RLS Prevalence

**FIGURE 2:**
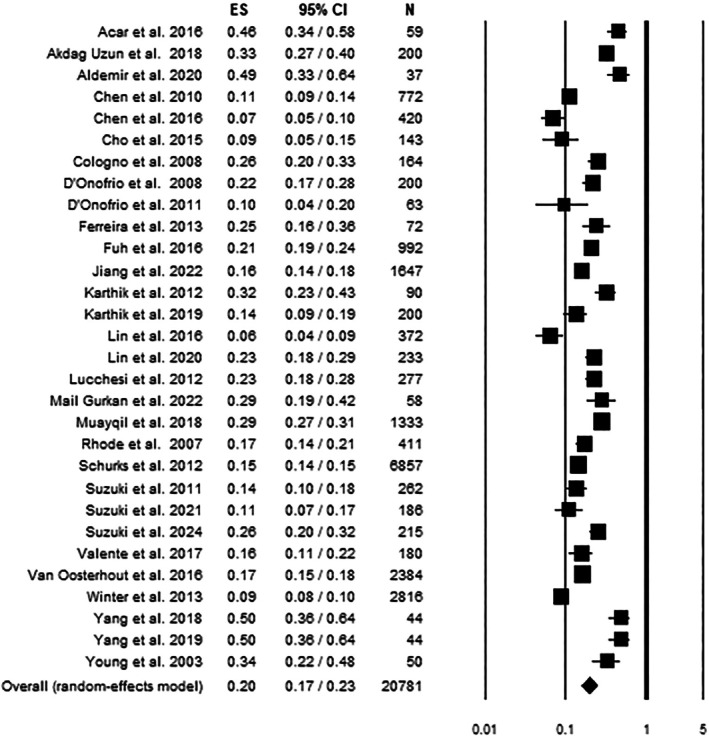
Forest plot for prevalence rate of Migraine + RLS. CI = confidence interval; ES = effect size; *N* = sample size.

The pooled prevalence of RLS among 20,781 individuals with migraine (MwoA, MA and CM) across 30 studies was 20% (95% CI: 17–23). Heterogeneity was high and statistically significant (*Q*(29) = 598.79, *p* < 0.001; *I* ^2^ = 95.16). No evidence of publication bias emerged (Egger's test: *p* = 0.113). A leave‐one‐out sensitivity analysis confirmed the robustness of the findings, as the exclusion of any single study did not substantially alter the overall prevalence estimate (see Table 2a and Figure 2).

**TABLE 2:** Summary of the results of meta‐analysis on prevalence, sociodemographic, clinical and behavioural migraine‐related features associated with the occurrence of RLS.

Outcomes	*K*	*N*	Pooled effect size (*p*)	(95% confidence intervals)		Homogeneity statistics					Egger's *t* test for publication bias	Trim and fill (estimated effect size)
				LL	UL	*Q* (df)	*p*	*I* ^2^	*τ* ^2^	*τ*		
(a) Prevalence	30	20,781	0.20 (**< 0.001**)	0.17	0.23	598.79(29)	**< 0.001**	95.16	0.25	0.50	1.64 (0.113)	0
(b) Sociodemographic aspects												
Age	15	3698	0.22 (**0.019**)	0.04	0.41	51.90 (14)	**< 0.001**	73.02	0.08	0.29	1.16 (0.267)	0
Sex	13	3221	0.84 (0.097)	0.69	1.03	5.83 (12)	0.925	0	0	0	−0.97 (0.345)	0
(c) Migraine‐related clinical aspects												
Age at onset	3	300	0.32 (**0.046**)	0.01	0.63	2.37 (2)	0.306	15.55	0.02	0.12	−0.27 (0.833)	0
Migraine duration	7	915	−0.19 (0.413)	−0.65	0.27	40.69 (6)	**< 0.001**	85.26	0.31	0.56	−1.82 (0.128)	2 [−0.19 (0.413)]
MA subtype	13	4663	1.36 (**0.009**)	1.08	1.72	41.91 (12)	**< 0.001**	71.51	0.10	0.32	1.65 (0.127)	0
MwoA subtype	10	4435	0.92 (0.067)	0.84	1.01	23.63 (9)	**0.005**	61.91	0.01	0.08	−2.21 (0.058)	0
CM subtype	5	2317	1.16 (**0.020**)	1.02	1.31	1.92 (4)	0.751	0	0	0	0.09 (0.936)	0
Migraine disability	7	2661	0.69 (**0.001**)	0.27	1.10	72.53 (6)	< **0.001**	91.73	0.26	0.51	1.35 (0.235)	0
Attack frequency	11	2977	0.22 (**< 0.001**)	0.13	0.32	6.70 (10)	0.753	0	0	0	−1.10 (0.301)	0
(d) Behavioural aspects												
Anxiety	5	733	0.63 (0.058)	−0.02	1.29	41.99 (4)	**< 0.001**	90.47	0.46	0.68	1.06 (0.365)	0
Depression	9	2286	0.61 (**< 0.001**)	0.38	0.84	26.14(8)	**0.001**	69.39	0.07	0.27	0.59 (0.573)	0
Sleep	9	5111	0.61 (**< 0.001**)	0.44	0.78	28.66(8)	**< 0.001**	72.09	0.04	0.20	2.77 (**0.028**)	1 [0.61 (< 0.001)]
Sensitivity analysis after removing Van Oosterhout et al. (2016)	8	2727	0.65 (**< 0.001**)	0.52	0.78	9.36(7)	0.228	25.19	0.01	0.09	1.26 (0.255)	0
Pain	3	260	0.45 (**0.005**)	0.13	0.77	0.56(2)	0.758	0	0	0	−6.60 (0.096)	0

*Note:* Statistically significant values are reported in bold.

Abbreviations: CM = chronic migraine; df = degrees of freedom; *K* = number of studies; LL = lower limit; MA = migraine with aura; MwoA = migraine without aura; *N* = total number of participants; *Q*, *I* ^2^, *τ* ^2^, *τ* = heterogeneity statistics; UP = upper limit.

#### 3.3.1 Moderator Analyses

Moderator analyses revealed significant effects for migraine duration (*k* = 11, *α* = 0, *B* = −0.06, *p* = 0.028) and migraine disability (*k* = 10, *α* = −0.56, *B* = −0.03, *p* = 0.022), suggesting that longer duration and greater disability were associated with increased RLS prevalence. Age also emerged as a significant moderator (*k* = 25, *α* = −0.07, *B* = −0.03, *p* = 0.047), indicating a positive association between higher age and the ES.

### 3.4 Meta‐Analytic Results on Demographic Aspects Associated With Migraine + RLS

A significant association was observed between the presence of RLS and age (*k* = 15, Hedges' *g* = 0.22, *p* = 0.019). Heterogeneity was substantial (*Q*(14) = 51.90, *p* < 0.001; *I* ^2^ = 73.02). No publication bias or trimmed studies were identified (*p* = 0.267; Table 2b). No significant association was found between RLS and sex (*k* = 13, RR = 0.84, *p* = 0.097), with low heterogeneity (*Q*(12) = 5.83, *p* = 0.925; *I* ^2^ = 0). Egger's test indicated no publication bias (*p* = 0.345) and no trimmed studies were detected (Table 2b). Education level was excluded from the analysis due to the limited number of studies reporting this variable. Forest plots for these demographic results are presented in Supporting Information Data S3.

### 3.5 Meta‐Analytic Results on Clinical Migraine Aspects Associated With Migraine + RLS

A significant association was found between RLS and MA subtype (*k* = 13, RR = 1.36, *p* = 0.009), with high heterogeneity (*Q*(12) = 43.91, *p* < 0.001; *I* ^2^ = 72.67) and no publication bias (*p* = 0.127) nor trimmed studies (Table 2c).

Conversely, no significant association emerged for MwoA subtype (*k* = 10, RR = 0.92, *p* = 0.067), despite high heterogeneity (*Q*(9) = 23.63, *p* = 0.005; *I* ^2^ = 61.91) and no publication bias (*p* = 0.058) or trimmed studies (Table 2c).

However, CM was significantly associated with RLS (*k* = 5, RR = 1.16, *p* = 0.020), with no heterogeneity (*Q*(4) = 1.92, *p* = 0.751; *I* ^2^ = 0) and no evidence of publication bias (*p* = 0.936) or trimmed studies (Table 2c).

A significant association was also found between RLS and age at migraine onset (*k* = 3, Hedges' *g* = 0.32, *p* = 0.046), with low and nonsignificant heterogeneity (*Q*(2) = 2.37, *p* = 0.306; *I* ^2^ = 15.55), with neither publication bias (*p* = 0.833) nor trimmed studies (Table 2c).

No significant association was observed between RLS and migraine duration (*k* = 7, Hedges' *g* = −0.19, *p* = 0.413). Heterogeneity was high (*Q*(6) = 40.69, *p* < 0.001; *I* ^2^ = 85.26), there was no publication bias (*p* = 0.128) and two trimmed studies were identified (*p* = 0.128; Table 2c).

A significant association was found between RLS and migraine disability (*k* = 7, Hedges' *g* = 0.69, *p* = 0.001), with high heterogeneity (*Q*(6) = 72.53, *p* = 0.001; *I* ^2^ = 91.73) and no evidence of publication bias (*p* = 0.235) nor trimmed studies (Table 2c).

RLS was also significantly associated with higher migraine attack frequency (*k* = 11, Hedges' *g* = 0.22, *p* < 0.001), with no heterogeneity (*Q*(10) = 6.70, *p* = 0.753; *I* ^2^ = 0) and there was no publication bias (*p* = 0.301) nor trimmed studies (Table 2c). Forest plots for these clinical outcomes are shown in Supporting Information Data S3.

### 3.6 Meta‐Analytic Results on Behavioural Aspects Associated With Migraine + RLS

RLS was significantly associated with depressive symptoms (*k* = 9, Hedges' *g* = 0.61, *p* < 0.001), with moderate heterogeneity (*Q*(8) = 26.14, *p* < 0.001; *I* ^2^ = 69.39) and there was no publication bias (*p* = 0.573) nor trimmed studies (Table 2d).

The association between RLS and anxiety symptoms was not statistically significant (*k* = 5, Hedges' *g* = 0.63, *p* = 0.058), with high heterogeneity (*Q*(4) = 41.99, *p* < 0.001; *I* ^2^ = 90.47) and no evidence of publication bias (*p* = 0.365) nor trimmed studies (Table 2d).

A significant association was found between RLS and pain intensity (*k* = 3, Hedges' *g* = 0.45, *p* = 0.005), with no heterogeneity (*Q*(2) = 0.56, *p* = 0.758; *I* ^2^ = 0) and no publication bias (*p* = 0.096) nor trimmed studies (Table 2d).

A significant association also emerged between RLS and poor sleep quality (*k* = 8, Hedges' *g* = 0.61, *p* < 0.001), with high heterogeneity (*Q*(8) = 28.66, *p* < 0.001; *I* ^2^ = 72.09). Egger's regression test revealed publication bias (*p* = 0.028) and one trimmed study was identified. Sensitivity analysis suggested the exclusion of Van Oosterhout et al. (2016), which resulted in a robust ES (*g* = 0.65), low heterogeneity (*I* ^2^ = 25.19) and no publication bias (*p* = 0.255). Forest plots for behavioural outcomes are presented in Supporting Information Data S3.

## 4 Discussion

This meta‐analysis aimed to investigate the aggregate prevalence and demographic, clinical and behavioural aspects associated with Migraine + RLS. Our findings revealed a pooled prevalence of 20% (95% CI: 17–23), with moderation analysis indicating that migraine disability, duration and age significantly affect the relationship between migraine and RLS. The prevalence of RLS among individuals with migraine is substantially higher than that observed in the general population (5%–10%; Allen et al. 2014; Gossard et al. 2021) and closely mirrors the rate reported in individuals with Parkinson's disease (Maggi et al. 2024). This finding highlights meaningful comorbidity and emphasises the need for routine RLS screening in migraineurs, particularly those with more severe disease courses.

Individuals with Migraine + RLS presented a more disabling migraine phenotype, characterised by higher attack frequency, greater pain intensity and higher migraine disability scores, aligning with the hypothesis of a central sensitisation model in which chronic nociceptive input and sleep dysregulation amplify pain processing and emotional reactivity (Haack et al. 2020; Harte et al. 2018).

The association between migraine and RLS appears biologically plausible when considering shared pathophysiological and clinical features. Specifically, dopaminergic dysfunction may represent a shared neurobiological substrate. In RLS, abnormalities in the A11 dopaminergic pathway, which projects to the neocortex, are thought to alter sensory processing, contributing to the hallmark sensorimotor restlessness (Clemens et al. 2006). In migraine, dopaminergic involvement is evidenced by the frequent presence of premonitory symptoms, such as yawning, nausea, vomiting and gastrointestinal disturbances, which typically precede headache onset and reflect central dopaminergic hypersensitivity (Charbit et al. 2010). These observations support the hypothesis that dopaminergic system dysregulation may underline the co‐occurrence of migraine and RLS (Cologno et al. 2008) and reinforce the need for further investigation into shared mechanisms with clinical implications for treatment. For instance, individuals with migraine frequently rely on chronic medication use, particularly drugs affecting dopaminergic pathways (e.g., flunarizine, paroxetine, sertraline), which may trigger or exacerbate RLS. There is a complex interplay between the pharmacological treatments of RLS and migraine. On the one hand, dopaminergic agonists commonly used for RLS may worsen migraine symptoms; on the other, certain medications prescribed for migraines, such as flunarizine, paroxetine and sertraline, can exacerbate RLS symptoms (Cannon and Larner 2011). Hence, in clinical practice, selecting migraine preventive therapies should consider their dopaminergic effects; alpha‐2‐delta ligands (i.e., gabapentin, pregabalin) may represent a preferable option for treating both conditions simultaneously (Silvestro et al. 2023).

Iron dysregulation represents another potential mechanism: while iron deficiency has been consistently reported in individuals with RLS (Trenkwalder and Paulus 2010), increased brain iron deposition has been observed in individuals with migraine, particularly, in regions such as the periaqueductal grey and appears to correlate with the frequency of attacks (Kruit et al. 2009).

Our analyses revealed that age, migraine onset, disability and attack frequency significantly influenced the migraine + RLS relationship. Older age was associated with increased comorbidity, possibly due to age‐related dopaminergic dysregulation or accumulated disease burden (Fila et al. 2023; Whittom et al. 2007). In contrast, sex was not significantly associated with RLS prevalence, suggesting that shared neurobiological vulnerabilities may be more critical than sex‐specific factors (Chen et al. 2010; Schürks et al. 2012).

Notably, RLS was significantly more common among individuals with MA and CM, but not among those with MwoA, supporting the involvement of cortical hyperexcitability and thalamocortical dysrhythmia in the comorbidity (Altamura et al. 2021; Goadsby et al. 2017). These findings are in line with studies demonstrating a stronger link between the MA subtype and RLS (Acar et al. 2016; Lin et al. 2020) and possibly explained by the cortical spreading depression model, which suggests neuronal depolarisation and wave‐like glial propagation, mechanisms thought to be shared by both conditions (Acar et al. 2016).

Furthermore, our results suggest that in individuals with MwoA, the association with RLS may not be apparent during the early or episodic stages. However, if migraine attacks become more frequent and the disorder progresses towards a chronic course, the comorbidity with RLS becomes more pronounced (Lucchesi et al. 2012), indicating that disease chronification may reveal or amplify shared neurophysiological mechanisms.

Behavioural and clinical features also played a central role. Depression, poor sleep quality and greater pain were all significantly associated with the Migraine+RLS phenotype. Depression may either result from or contribute to RLS, through mechanisms such as impaired sleep, low physical activity and poor nutrition or may share underlying pathophysiological substrates (Hornyak 2010; Picchietti and Winkelman 2005). It is therefore essential to carefully assess the presence of comorbid depressive disorders, not only to implement preventive strategies that address both conditions in conjunction with sleep hygiene and regular psychological support, but also to guide more cautious and informed use of antidepressant medications. This is particularly important given that certain antidepressants, especially selective serotonin reuptake inhibitors (SSRIs), may exacerbate or increase the risk of RLS.

Interestingly, anxiety was not significantly associated, suggesting that the affective profile of this comorbidity is more somatic‐depressive than anxious‐hyperaroused in nature (Kim et al. 2013; Xu et al. 2022).

RLS is frequently associated with various sleep disturbances, particularly, insomnia and periodic limb movements (PLMS), which are highly prevalent among RLS patients and contribute to poor sleep efficiency and increased arousal (Becker and Novak 2014; Bjorvatn et al. 2005). In fact, up to 85% of RLS patients report significant insomnia and recent evidence links RLS with other sleep disorders such as sleep apnea syndrome, with a prevalence of 21% (Pistorius et al. 2020). This link further strengthens the argument for integrated management approaches that address sleep and mood disturbances in migraineurs with RLS.

Pain is another critical dimension in the RLS‐migraine overlap. Many RLS patients report painful sensations, though often not easily described using conventional pain terminology, which can intensify with poor sleep and fatigue (Winkelman et al. 2013). This is particularly important considering the overlap between RLS and other chronic pain conditions such as fibromyalgia and rheumatoid arthritis (Yunus and Aldag 1996). The dopaminergic system appears to play a central role in the pathophysiological overlap.

Despite robust findings, several methodological limitations must be acknowledged. Significant heterogeneity was observed, especially in behavioural outcomes, partly due to the use of varying RLS diagnostic tools and inconsistent measurement standards across studies (e.g., Cho et al. 2015; Muayqil et al. 2018; Karthik et al. 2012). Moreover, although sensitivity analyses confirmed the robustness of key findings, certain associations, especially regarding sleep, were influenced by publication bias (e.g., Van Oosterhout et al. 2016).

Finally, only a limited number of studies have evaluated the association between RLS and migraine clinical subtypes, based on the International Classification of Headache Disorders third edition (ICHD‐3; Olesen 2018); further studies should systematically investigate this relationship across different migraine subtypes to clarify distinct patterns of comorbidity.

## 5 Conclusion

Clinically, these findings underscore the importance of screening for RLS in migraineurs, especially those with CM or MA subtype, poor sleep or comorbid depression. Pharmacological strategies should avoid agents that worsen RLS and favour those that can potentially alleviate both conditions. Non‐pharmacological interventions, including regular physical exercise, sleep hygiene and psychological therapies, may enhance treatment outcomes. Future longitudinal and neuroimaging studies should further explore the temporal and mechanistic links between migraine and RLS, including the role of neurotransmitter systems like glutamate and adenosine, to identify potential therapeutic targets.

In conclusion, this meta‐analysis confirms a strong association between RLS and migraine, particularly, in individuals with MA, CM and those experiencing greater pain and affective burden. These findings emphasise the clinical importance of screening for RLS in migraine management and point to shared neurobiological pathways that warrant further investigation.

## Author Contributions

**Florindo d'Onofrio:** conceptualization, writing – review and editing, supervision. **Maria Cropano:** investigation, writing – original draft, methodology, formal analysis, data curation. **Giada Panzino:** investigation, writing – original draft, methodology, formal analysis, data curation. **Mariachiara Gaita:** investigation, methodology, data curation, formal analysis. **Giulio Cicarelli:** writing – review and editing. **Piero Barbanti:** writing – review and editing. **Gerardo Casucci:** writing – review and editing. **Simona Raimo:** conceptualization, investigation, writing – original draft, methodology, formal analysis, data curation, supervision. **Antonio Costanzo:** investigation, conceptualization, writing – original draft, methodology, formal analysis, data curation, supervision.

## Ethics Statement

The authors have nothing to report.

## Consent

The authors have nothing to report.

## Conflicts of Interest

The authors declare no conflicts of interest.

## Supporting information


**Data S1:** Supporting Information.

**Data S2:** Supporting Information.

**Data S3:** Supporting Information.

## Data Availability

The data that support the findings of this study are available in the Supporting Information of this article.
