# Supplementary material for: Migraine and Restless Legs Syndrome: A Meta‐Analysis
Source: J Sleep Res. 2025 Sep 19;35(3):e70202. doi: 10.1111/jsr.70202 (PMC13193476; doi:10.1111/jsr.70202)
Supplement: Supplementary file 1 — Data S1: Supporting Information. [file JSR-35-e70202-s002.docx]

Supplementary Material 1. Quality assessment of studies in the meta-analysis using the Modified Version of the Quality Assessment of Diagnostic Accuracy Studies (QUADAS).

| **Authors** | **QS ASSESSMENT** | | | | |
| --- | --- | --- | --- | --- | --- |
|  | **QS-1:**  **Patient Selection** | **QS-2:**  **Index Test** | **Q3-3:**  **Reference Standard** | **QS-4:**  **Flow and Timing** | **QS TOT** |
| Acar et al., 2016 | 5 | 7 | 5 | 1 | 18 |
| Akdag Uzun et al., 2018 | 6 | 6 | 5 | 0 | 17 |
| Aldemir et al., 2020 | 6 | 6 | 5 | 0 | 17 |
| Chen et al., 2010 | 6 | 6 | 5 | 1 | 18 |
| Chen et al.,2016 | 6 | 6 | 5 | 1 | 18 |
| Cho et al., 2015 | 5 | 6 | 4 | 1 | 16 |
| Cologno et al., 2008 | 6 | 7 | 4 | 0 | 17 |
| D’Onofrio et al., 2008 | 6 | 7 | 5 | 0 | 18 |
| D’Onofrio et al., 2011 | 6 | 7 | 4 | 1 | 18 |
| Ferreira et al., 2013 | 6 | 6 | 5 | 0 | 17 |
| Fuh et al., 2016 | 6 | 6 | 4 | 1 | 17 |
| Jiang et al., 2022 | 6 | 6 | 3 | 0 | 15 |
| Karthik et al., 2012 | 6 | 6 | 4 | 1 | 17 |
| Karthik et al., 2019 | 6 | 7 | 5 | 1 | 19 |
| Lin et al., 2016 | 6 | 7 | 4 | 1 | 18 |
| Lin et al., 2020 | 6 | 6 | 5 | 1 | 18 |
| Lucchesi et al., 2012 | 6 | 7 | 4 | 1 | 18 |
| Mail Gurkan et al., 2022 | 5 | 6 | 4 | 1 | 16 |
| Muayqil et al., 2018 | 5 | 6 | 4 | 0 | 15 |
| Rhode et al., 2007 | 6 | 5 | 5 | 0 | 16 |
| Schurks et al., 2012 | 5 | 6 | 4 | 1 | 16 |
| Suzuki et al., 2011 | 6 | 6 | 4 | 1 | 17 |
| Suzuki et al., 2021 | 6 | 6 | 5 | 1 | 18 |
| Suzuki et al., 2024 | 6 | 6 | 5 | 0 | 17 |
| Valente et al., 2017 | 6 | 7 | 4 | 1 | 18 |
| Van Oosterhout et al., 2016 | 6 | 7 | 4 | 0 | 17 |
| Winter et al., 2013 | 6 | 5 | 4 | 0 | 15 |
| Yang et al., 2018 | 6 | 6 | 5 | 0 | 17 |
| Yang et al., 2019 | 6 | 6 | 5 | 0 | 17 |
| Young et al., 2003 | 6 | 6 | 4 | 1 | 17 |

Abbreviations: QS= Quality score; QS TOT= Total Quality score.
