# Supplementary material for: Migraine and Restless Legs Syndrome: A Meta‐Analysis
Source: J Sleep Res. 2025 Sep 19;35(3):e70202. doi: 10.1111/jsr.70202 (PMC13193476; doi:10.1111/jsr.70202)
Supplement: Supplementary file 2 — Data S2: Supporting Information. [file JSR-35-e70202-s001.docx]

Supplementary Material 2. Migraine-related behavioural features, of the Migraineurs population in the included studies.

| Authors | Migraine+RLS patients | | | | Migraine-RLS patients | | | |
| --- | --- | --- | --- | --- | --- | --- | --- | --- |
|  | Dep | Anx | Sleep Quality | Pain | Dep | Anx | Sleep Quality | Pain |
| Acar et al., 2016 | 18.3±8.9  (Beck-D) | 26.7±12.8  (Beck-A) | 11.1±3.7  (PSQI) | 7.0±2.0  (VAS) | NR | NR | NR | 5.9±1.6  (VAS) |
| Akdag Uzun et al., 2018 | NR | 36.2±14.4  (BAI) | NA | NA | NR | 32.8±12.6  (BAI) | NA | NA |
| Aldemir et al., 2020 | 22.3±2.7  (BDI) | 22.6±3.3  (BAI) | NR | NR | 15.8± 2.6  (BDI) | 11.9±2.5  (BAI) | NR | NR |
| Chen et al., 2010 | NR | NR | 11.1±4.1  (PSQI) | NA | NR | NR | 8.9±4.0  (PSQI) | NA |
| Chen et al., 2016 | NA | NA | NA | NA | NA | NA | NA | NA |
| Cho et al., 2015 | NA | NA | NA | 6.8±2.2  (VAS) | NA | NA | NA | 6.1±1.9  (VAS) |
| Cologno et al., 2008 | NR | NR | NR | NR | NR | NR | NR | NR |
| D’Onofrio et al., 2008 | NR | NR | NA | NR | NR | NR | NA | NR |
| D’Onofrio et al., 2011 | 6.2 ± 5.5  (HDRS) | 5.2 ± 3.5  (HAMA) | NA | NA | 6.1±5.0  (HDRS) | 6.2±3.0  (HAMA) | NA | NA |
| Ferreira et al., 2013 | NR | NA | NA | NA | NR | NA | NA | NA |
| Fuh et al., 2016 | 14.0±8.5  (BDI) | NA | 10.7±4.1  (PSQI) | NA | 9.9±7.8  (BDI) | NA | 8.3±3.9  (PSQI) | NA |
| Jiang et al., 2022 | NA | NA | NA | NA | NA | NA | NA | NA |
| Karthik et al., 2012 | NA | NA | NR | NA | NA | NA | NR | NA |
| Karthik et al., 2019 | 4.14±3.24  (HADS-D) | 4.3±3.6  (HADS-A) | 3.3±2.6  (PSQI) | NA | 3.0±2.9  (HADS-D) | 4.0±3.5 (HADS-A) | 2.2±2.0  (PSQI) | NA |
| Lin et al., 2016 | NR | NR | NR | NA | NR | NR | NR | NA |
| Lin et al., 2020 | 15.6±8.70  (BDI) | 7.6±3.95  (HADS-A) | 11.8±4.1  (PSQI) | NA | 8.5±8.5  (BDI) | 6.1±4.5  (HADS-A) | 9.3±3.9  (PSQI) | NA |
| Lucchesi et al., 2012 | NA | NA | NR | NA | NA | NA | NR | NA |
| Mail Gurkan et al., 2022 | NA | NA | NA | 9.1±0.7  (VAS) | NA | NA | NA | 8.7±1.3  (VAS) |
| Muayqil et al., 2018 | NA | NA | NA | NA | NA | NA | NA | NA |
| Rhode et al., 2007 | 12.1±7.7  (BDI) | NA | NA | NA | 8.0±7.3  (BDI) | NA | NA | NA |
| Schurks et al., 2012 | NR | NA | NA | NA | NR | NA | NA | NA |
| Suzuki et al., 2011 | 18.9 ± 11.0  (BDI-II) | NA | 8.4 ± 3.6  (PSQI)  10.8 ± 4.9  (ESS) | NA | 12.2±9.2  (BDI-II) | NA | 5.2±3.1  (PSQI)  8.1±4.8  (ESS) | NA |
| Suzuki et al., 2021 | NA | NA | NA | NR | NA | NA | NA | NR |
| Suzuki et al., 2024 | NA | NA | NR | NA | NA | NA | NR | NA |
| Valente et al., 2017 | NA | NA | 5.3±2.5  (PSQI)  5.7±3.9  (ESS) | NA | NA | NA | 4.0±2.6  (PSQI)  5.7±3.8  (ESS) | NA |
| Van Oosterhout et al., 2016 | NR | NA | 7.4±3.7  (PSQI) | NA | NR | NA | 6.3±3.6  (PSQI) | NA |
| Winter et al., 2013 | NR | NA | NA | NA | NR | NA | NA | NA |
| Yang et al., 2018 | 9.1±5.3  (BDI) | NR | 10.5±4.0  (PSQI) | NA | 8.4±5.9  (BDI) | NR | 7.9±3.1  (PSQI) | NA |
| Yang et al., 2019 | 13.9±8.7  (BDI) | NR | 11.2±3.6  (PSQI) | NA | 9.4±7.8  (BDI) | NR | 6.9±3.1  (PSQI) | NA |
| Young et al., 2003 | NA | NA | NA | NA | NA | NA | NA | NA |

Abbreviations: RLS=Restless legs syndrome; Migraine+RLS= patients with migraine and RLS; Migraine-RLS= patients with migraine without RLS; NR= Not Reported; NA= Not Assessed; Dep= Depression; Anx= Anxiety; Beck-A= Beck Anxiety Inventory; Beck-D= Beck Depression Inventory; PSQI= Pittsburgh Sleep Quality Index; VAS= Visual Analog Scale; BDI= Beck Depression Inventory; BDI-II= Beck Depression Inventory-II; BAI= Beck Anxiety Inventory; HDRS= Hamilton Depression Rating Scale; HAMA= Hamilton Anxiety Rating Scale; HADS= Hospital Anxiety and Depression Scale; HADS-A= HADS anxiety; HADS-D= HADS depression; ESS= Epworth Sleepiness Scale.
