# Supplementary material for: Migraine and Restless Legs Syndrome: A Meta‐Analysis
Source: J Sleep Res. 2025 Sep 19;35(3):e70202. doi: 10.1111/jsr.70202 (PMC13193476; doi:10.1111/jsr.70202)
Supplement: Supplementary file 3 — Data S3: Supporting Information. [file JSR-35-e70202-s003.docx]

Supplementary Material 3. Forest plots for demographic, clinical and behavioral aspects.


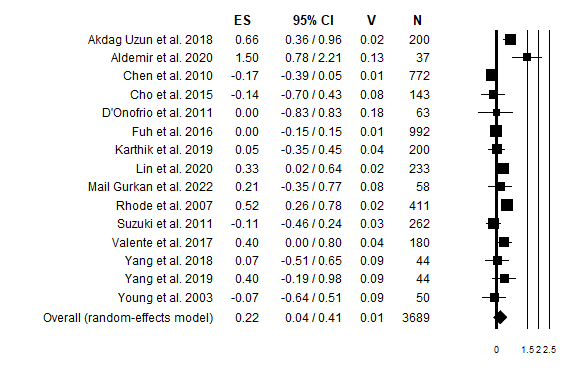


Forest plot showing effect sizes (Hedges’ g) and 95% confidence intervals (CIs) comparing age between Migraine-RLS and Migraine+RLS. ES, effect size; CI, confidence interval; V, Variance; N, total number; RLS, Restless Legs Syndrome.


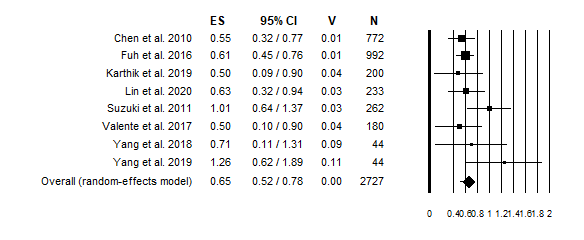


Forest plot showing effect sizes (Hedges’ g) and 95% confidence intervals (CIs) comparing sleep between Migraine-RLS and Migraine+RLS. ES, effect size; CI, confidence interval; V, Variance; N, total number; RLS, Restless Legs Syndrome.


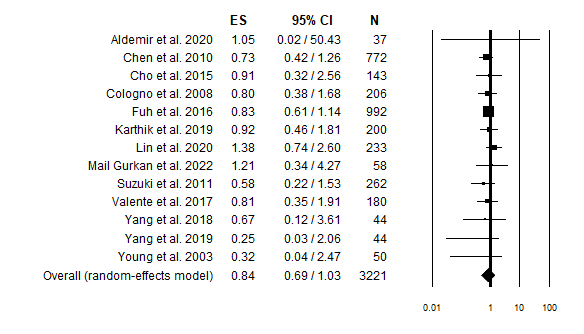


Forest plot showing effect size (Risk Ratio) and 95% confidence intervals (CIs) comparing the effect of sex (number of male patients) between Migraine-RLS and Migraine+RLS. ES, effect size; CI, confidence interval; N, total number; RLS, Restless Legs Syndrome.


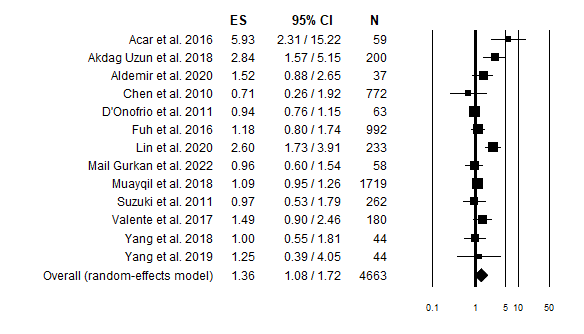


Forest plot showing effect size (Risk Ratio) and 95% confidence intervals (CIs) comparing the effect of migraine subtype Aura (MA) between Migraine-RLS and Migraine+RLS. ES, effect size; CI, confidence interval; N, total number; RLS, Restless Legs Syndrome.


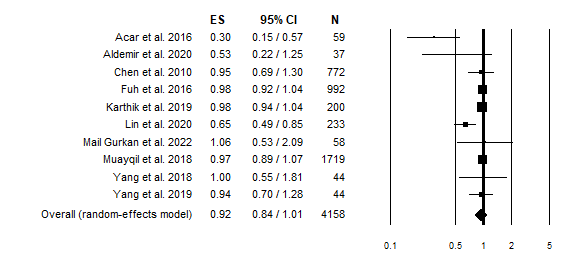


Forest plot showing effect size (Risk Ratio) and 95% confidence intervals (CIs) comparing the effect of migraine subtype without Aura (MwoA) between Migraine-RLS and Migraine+RLS. ES, effect size; CI, confidence interval; N, total number; RLS, Restless Legs Syndrome.


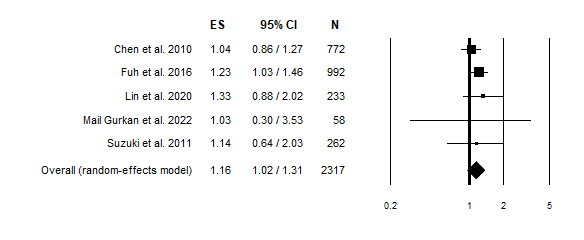


Forest plot showing effect size (Risk Ratio) and 95% confidence intervals (CIs) comparing the effect of migraine subtype Chronic (CM) between Migraine-RLS and Migraine+RLS. ES, effect size; CI, confidence interval; N, total number; RLS, Restless Legs Syndrome.


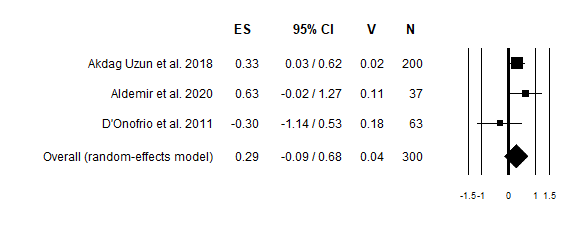


Forest plot showing effect sizes (Hedges’ g) and 95% confidence intervals (CIs) comparing migraine onset between Migraine-RLS and Migraine+RLS. ES, effect size; CI, confidence interval; V, Variance; N, total number; RLS, Restless Legs Syndrome.


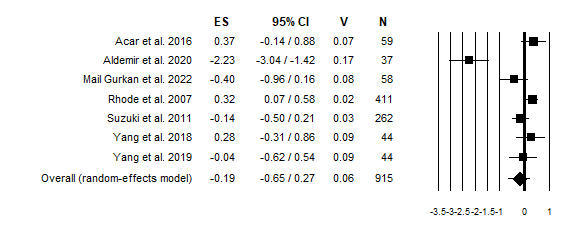


Forest plot showing effect sizes (Hedges’ g) and 95% confidence intervals (CIs) comparing migraine duration between Migraine-RLS and Migraine+RLS. ES, effect size; CI, confidence interval; V, Variance; N, total number; RLS, Restless Legs Syndrome.


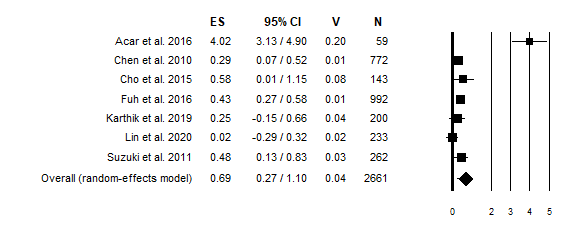


Forest plot showing effect sizes (Hedges’ g) and 95% confidence intervals (CIs) comparing migraine disability between Migraine-RLS and Migraine+RLS. ES, effect size; CI, confidence interval; V, Variance; N, total number; RLS, Restless Legs Syndrome.


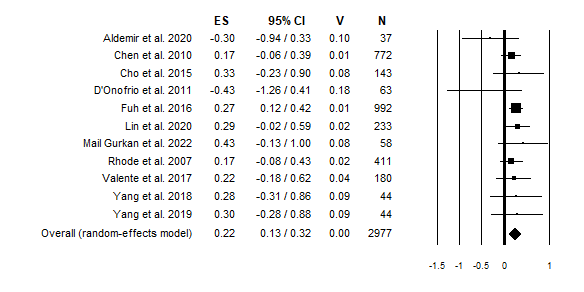


Forest plot showing effect sizes (Hedges’ g) and 95% confidence intervals (CIs) comparing frequency of migraine attacks between Migraine-RLS and Migraine+RLS. ES, effect size; CI, confidence interval; V, Variance; N, total number; RLS, Restless Legs Syndrome.


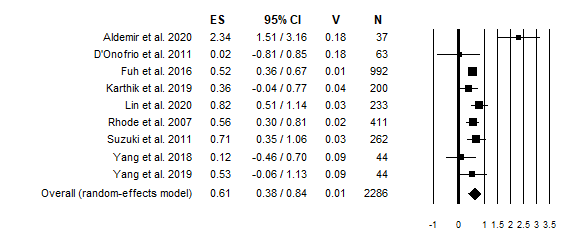


Forest plot showing effect sizes (Hedges’ g) and 95% confidence intervals (CIs) comparing depression between Migraine-RLS and Migraine+RLS. ES, effect size; CI, confidence interval; V, Variance; N, total number; RLS, Restless Legs Syndrome.


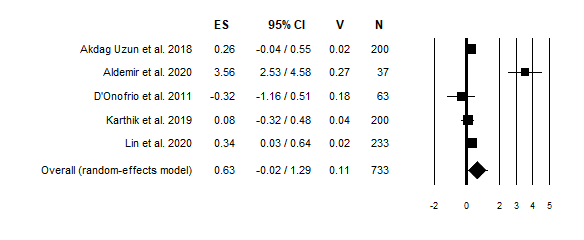


Forest plot showing effect sizes (Hedges’ g) and 95% confidence intervals (CIs) comparing anxiety between Migraine-RLS and Migraine+RLS. ES, effect size; CI, confidence interval; V, Variance; N, total number; RLS, Restless Legs Syndrome.


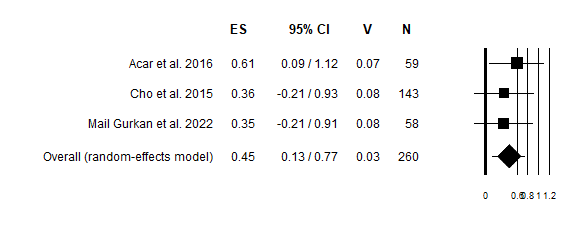


Forest plot showing effect sizes (Hedges’ g) and 95% confidence intervals (CIs) comparing pain between Migraine-RLS and Migraine+RLS. ES, effect size; CI, confidence interval; V, Variance; N, total number; RLS, Restless Legs Syndrome.
